# A clinical study of immediate postoperative corneal edema in patients undergoing minor incision cataract surgery in a teaching hospital

**DOI:** 10.22336/rjo.2024.69

**Published:** 2024

**Authors:** Anusha Aynala, Thanuja Gopal Pradeep

**Affiliations:** Department of Ophthalmology, Ramaiah Medical College, Ramaiah University of Applied Sciences, Bangalore, India

**Keywords:** corneal edema, Descemet’s membrane detachment, SICS, AS-OCT = Anterior segment Optical coherence Tomography, BCVA = Best corrected visual acuity, CCT = Central corneal thickness, DM = Descemet’s membrane, DMD = Descemet’s membrane detachment, HELP = Height, Extent, Length, Pupil, ICE = Irido corneal endothelial syndrome, IOL = Intraocular lens, MSICS = Manual small incision cataract surgery, No. = Number, NS = Nuclear sclerosis, OCT = Optical Coherence Tomography, PCIOL = Posterior chamber intraocular lens, SD = Standard deviation, SICS = Small Incision Cataract Surgery, SPSS = Statistical Package for the Social Sciences, VA = Visual acuity

## Abstract

**Background:**

Transient corneal edema is one of the most common complications observed after cataract surgery. If the center of the cornea is involved, it may result in impaired visual acuity in the immediate postoperative period. Hence, it concerns both the surgeon and the patient. Descemet’s membrane detachment (DMD) is a less recognized cause of immediate corneal edema that can lead to long-term endothelial failure. Immediate recognition and surgical management may preserve vision in these patients.

**Objectives:**

To determine the proportion of corneal edema in subjects undergoing manual minor incision cataract surgery, grade them under slit-lamp examination and anterior segment optical coherence tomography, and correlate the findings.

**Materials and methods:**

We included patients who underwent manual small-incision cataract surgery (SICS) in the Department of Ophthalmology of a teaching hospital from November 2019 to May 2021. Postoperatively, all patients were subjected to detailed ophthalmic evaluation, and those with corneal edema underwent anterior segment OCT to determine the corneal edema and status of Descemet’s membrane.

**Results:**

Out of 922 patients who underwent manual SICS, 91 patients (9%) had corneal edema; the mean corneal thickness in the area of corneal edema on AS-OCT was found to be 726.92µm with an SD of 137.00µm and the mean CCT was 497.55 with an SD of 49.70. Seven patients (7.69%) had Descemet’s membrane detachment (DMD) postoperatively, and the mean DMD at the highest point was 140.76µm. Five patients recovered with medical management; two were treated with anterior chamber air injection.

**Discussions:**

Our study showed 9% corneal edema on postoperative day one, lower than other studies (18-44%). Diabetes mellitus type 2 was not associated with corneal edema in SICS cases, contrary to findings in phacoemulsification as reported in other studies. Pupillary manipulation was observed in only 2.2% of the cases, not being a risk factor. Surgeon experience significantly affected corneal edema, with trainee surgeons reporting more cases (44.5%, P=0.004). Hard cataracts (71.4%) caused higher endothelial damage, particularly in nuclear sclerosis grade NS5 (27.47%). The study underscores planning surgery based on cataract hardness, surgeon expertise, and proper intraoperative techniques to minimize complications.

**Conclusion:**

Postoperative corneal edema following cataract surgery is a known complication usually resolved by medical management. More excellent surgical experiences with a shorter duration of surgery and proper instrumentation were associated with reduced early postoperative corneal edema. Early detection and classification of DMD with the help of AS-OCT for those requiring medical and surgical management aid recognize the clinically relevant DMD. Thus, a timely switch to surgical management helps maintain vision in patients with DMD.

## Introduction

Corneal edema is the condition of excess corneal hydration caused by altered fluid transport across the cornea [1] when there is a break in the anatomic or functional integrity of the corneal epithelium or endothelium. Among the several complications that might occur in cataract surgery, corneal edema is one of the most frequently encountered complications. Corneal edema may occur due to endothelial pump failure following surgery, which may be due to mechanical injury, chemical injury, subsequent infection/inflammation, or concurrent/preexisting endothelial compromise [2]. The present study aims to determine the proportion of corneal edema in subjects undergoing manual minor incision cataract surgery, grade them under slit-lamp examination and anterior segment optical coherence tomography, and correlate the findings.

## Materials and methods

An observational study was conducted on patients undergoing minor incision cataract surgery in the Department of Ophthalmology of a Teaching Institute from Nov 2019 to May 2021. All patients aged more than 40 years, undergoing manual small incision cataract surgery (MSICS) were included in the study. Patients with pre-existing corneal opacities, iridocorneal endothelial (ICE) syndrome, Fuchs’ endothelial dystrophy, unhealthy cornea before surgery, eye trauma, acute glaucoma, inflammation of the eye, especially long-term or chronic inflammation, and traumatic cataract were excluded. After obtaining informed consent, demographics of all the patients who had corneal edema were collected, which included age, sex, and preoperative data such as the eye that was operated, systemic illnesses like diabetes mellitus and hypertension, intraocular pressures, maximum dilatation of the pupil, visual acuity (VA), and grade of cataract. Intraoperative data such as duration of surgery, the surgeon who operated (experienced or trainee surgeons), tunnel complications, pupil manipulations, posterior capsular rents, Descemet’s membrane detachment noted on the table, and type of intraocular lens used was recorded. Post-operative data on day one, such as best-corrected visual acuity (BCVA), intraocular pressures, zone involvement of corneal edema (central or periphery), slit-lamp grading of the corneal edema (as shown in **Table 1**) [3], corneal thickness on AS-OCT, central corneal thickness (CCT), any DMD, if present measurement of DMD at its maximum point was noted. AS-OCT (TOPCON 3D OCT-1 MAESTRO) was performed on all patients. If DMD was stressed, it was assessed based on the HELP algorithm described by Dhivya et al. [4].

**Table 1:** Slit lamp grading of corneal edema: adapted from Strass et al.

0	NONE	The cornea is transparent and clear
1	MILD	Dull glass epithelium, which may include micro-optic vacuoles
2	MODERATE	Dull glass epithelium and stroma with large numbers of vacuoles
3	SEVERE	Epithelial bullae and/or marked stromal edema (grayish-white translucency

### Sample size

The sample size was calculated based on a previous study conducted by Tarafder et al., in which 18% of the subjects had corneal edema in subjects who underwent minor incision cataract surgery. The present research expected a similar result with a 95% confidence interval and 5% absolute precision. Thus, it required a minimum of 227 subjects [5].

### Statistical analysis

Data were described in terms of range, mean ± standard deviation (± SD), frequencies (number of cases), and relative frequencies (percentages) as appropriate. A comparison of quantitative variables between the study variables using a paired t-test. For comparing categorical data, the Chi-square (χ2) test was performed, and the exact test was used when the expected frequency was less than 5. Pearson Correlation was used. A probability value (p-value) less than 0.05 was considered statistically significant. All statistical calculations were done using (Statistical Package for the Social Science) SPSS 16 version (SPSS Inc., Chicago, IL, USA) statistical program for Microsoft Windows.

## Results

A total of 922 patients who underwent manual minor incision cataract surgery in the Department of Ophthalmology between November 2019 and May 2021, conforming to the inclusion and exclusion criteria, were enrolled in this study. 91 out of 922 (9%) patients had corneal edema on postoperative day one. Out of 91 patients who had corneal edema, 50 (54.9%) were females, and 41 (45.1%) were males. The patients’ mean age was 52.99 ± 9.96 years (40-74 years). Only 6 (6.6%) patients had diabetes, and 6 (6.6%) were hypertensives.

Based on nuclear sclerosis, cataracts have been divided into soft and hard cataracts, i.e., nuclear sclerosis grades 1 and 2 (NS1, NS2) as soft cataracts and nuclear sclerosis grades 3, 4, and 5 (NS3, NS4, NS5) along with mature cataracts as hard cataracts. Soft cataracts were present in 26 (28.6%) patients, and hard cataracts were observed in 65 (71.4%) patients. Pseudoexfoliation was present in only 5 (5.5%) of the patients. Patients were divided into two groups based on maximum dilatation of the pupil, pupil dilatation greater than or equal to 7 mm, and those with less than 7 mm dilatation. Seventy-two patients, i.e., 72 (70.1%), were well dilating.

Out of the 91 patients with corneal edema, 39 (42.9%) were performed by junior residents, followed by associate and assistant professors, 17 (18.7%) each, and senior residents 10 (11.0%). We categorized surgeons into two groups based on experience, i.e., experienced and non-experienced surgeons. Even though an equal number of corneal edemas were seen in both the trainee (49 cases) and professional groups (42 cases), DMD was observed in 13 cases (86.6%) operated by trainee surgeons. Central and peripheral zones were equally affected by both trainee surgeons and experienced surgeons. There was no statistical difference between the groups to the zones involved (P-value > 0.949) by the Chi-square test.

Postoperatively, a maximum number of patients had Grade 1 corneal edema on slit-lamp examination, followed by Grade 2 and Grade 3, which were almost equal in proportion (**Table 2**).

**Table 2:** Proportion of corneal edema as graded on slit lamp examination

SLIT LAMP GRADING OF CORNEAL EDEMA	NO. OF CASES	PERCENTAGE
Grade 1	44	48.4%
Grade 2	24	26.4%
Grade 3	23	25.3%

The grade of cataracts in the zones involved is shown in **Table 3**. In our study, we found that the occurrence of corneal edema was directly proportional to the increase of nuclear sclerosis grade, i.e., corneal edema was observed highest in NS5 (27.47%) followed by NS3 (26.37%) and NS4 (23.08%), which was not statistically significant (P-value 0.31). Of 26 soft cataracts, 22 (84.6%) had central corneal edema, and out of 65 hard cataracts, 46 (70.7%) had central corneal edema. The central corneal edema in both groups was not statistically significant (P-value 0.187) by Fisher’s exact test.

**Table 3:** Grade of cataract to zones of corneal edema involved

GRADE	CENTRAL	PARACENTRAL	PERIPHERAL	TOTAL CORNEA	TOTAL NO. OF CASES	PERCENTAGE
**NS1**	2	1	0	1	4	4.40%
**NS2**	3	1	1	4	9	9.89%
**NS3**	14	3	5	2	24	26.37%
**NS4**	13	3	0	5	21	23.08%
**NS5**	15	3	3	4	25	27.47%
**MATURE CATARACT**	4	0	3	1	8	8.79%

57 (83.8%) of the patients with central corneal edema had CCT < 550 micrometers and 11 (16.2%) of the patients had CCT of > 550 micrometers, whereas 21 (91.3%) of the patients with peripheral corneal edema, had CCT > 550 micrometers. In our study, we measured corneal edema and CCT on AS-OCT. The mean corneal thickness was 726.92 with an SD of 137.00, and the mean CCT was 497.55 with an SD of 49.70. The total number of DMDs noted intraoperatively was fifteen, of which eight patients DM was found to be attached postoperatively as they were small DMDs, and seven patients had DMD on post-op day one as identified on AS-OCT. All seven cases were performed by trainee surgeons, which was statistically significant using the Chi-square test (P-value 0.04). Postoperative DMD was almost equally present in all cornea grades (**Table 4**).

**Table 4:** Correlation slit lamp grading of corneal edema and Descemet’s membrane detachment

Slit-lamp grading of corneal edema			Mean DMD
	Corneal edema	DMD	
GRADE 1	42	2	47µm
GRADE 2	22	3	86.3µm
GRADE 3	21	2	289µm

Hypertonic sodium chloride was used in 67 (72.8%) patients, and 24 (26.1%) patients with mild corneal edema required no medication, and edema resolved spontaneously.

PCIOL was used in 76 (82.6%) cases, out of which 56 (73.7%) had central corneal edema and 20 (26.3%) had edema peripherally. Iris claw was used in 15 (16.3%) of the patients, out of which 12 (80%) had central corneal edema, whereas 3 (20%) had edema peripherally. 70 (89.7%) of the patients who had PCIOL presented a CCT of < 550 micrometers, which was statistically significant when compared to patients who had iris-claw (P-value 0.001) by the Chi-square test.

Five patients with DMD were treated medically as they were small DMDs (mean DMD 86.3µm), and two patients were treated with air injection as their DMD was > 100µm (mean 289µm, following which the DMD was found to be attached to AS-OCT). Vision drastically improved in both patients who had undergone air injection for DMD. Visual acuity was better than 6/36 on POD1 after the air injection.

## Discussion

A total of 922 patients were included in our study, out of which 91 (9%) patients had corneal edema on post-op day one compared to a survey conducted by Kauser A et al. [6], which reported 44% of corneal edema. Tarfder et al. reported 18% of the cases [5]. The mean age of patients was 52.99 ± 9.96 years. Maximum patients (50.5%) belonged to the age group of 60-70 years. Though the existence of diabetes mellitus type 2 appears to be a significant risk factor for the development of corneal edema in phacoemulsification cases, as reported by Tsaousis KT et al. [7], we did not find any association between diabetes and corneal edema in SICS cases.

Kreku R et al. showed that mechanical pupillary dilatation can lead to corneal edema postoperatively [8]. In our study, only 2 (2.2%) patients had undergone pupillary manipulation intraoperatively, possibly due to the number of patients with poor pupillary dilatation.

In the present study, corneal edema was observed almost equally in both experienced (46.2%) and trainee surgeons (53.9%) (P-value > 0.01) because more cases, i.e., have been operated by the skilled surgeons, 812 out of 922, and only 110 by the trainee surgeons. In the trainee surgeons, corneal edema was statistically significant (49/110=44.5), and a maximum number of DMD was observed on AS-OCT post-operatively in trainee surgeons (P-value 0.004 using the Chi-square test), which was statistically significant. Surgeon experience may be crucial in preventing this early post-operative corneal edema by identifying and using techniques to reattach the DM intraoperatively. During the learning curve, a trainee doctor is more likely to use improper instrumentation and require prolonged surgery time.

In our study, out of all patients with corneal edema, 65 (71.4%) of the cases were hard cataracts, and 26 (28.6%) were soft cataracts. We found that a maximum number of corneal edemas was observed in patients with increased nuclear sclerosis grade, i.e., corneal edema was observed highest in NS5 (27.47%) followed by NS3 (26.37%) and NS4 (23.08%), which was similar to a study conducted by Mahdy MA et al. [9].

Of 26 soft cataracts, 22 (84.6%) had central corneal edema, probably because of the difficulty of the cortical wash. Of 65 hard cataracts, 46 (70.7%) had central corneal edema, probably due to endothelial damage during nucleus delivery. Central corneal edema in both groups was not statistically significant (P-value 0.187) and could occur equally in soft and complex cataracts. The surgeon needs to plan the surgery accordingly by doing an excellent hydrodissection to facilitate cortical wash in soft cataracts and using adequate viscoelastics in hard cataracts to prevent endothelial damage. There is no statistical difference between the zone involved and the surgeons (trainee and experienced), as the experienced surgeons do all cataracts with higher nuclear grades. It is known that endothelial cell loss is noted with increased nuclear hardness [9], and more hard cataract surgeries are done by experienced surgeons than trainee surgeons.

In the present study, we have graded corneal edema as mild, moderate, and severe on slit-lamp examination [3]. Moreover, we found that there was no statistically significant difference between the slit lamp grading of the cornea and the zone involved in CCT.

In addition, most patients with iris-claw had central corneal edema compared to patients with PCIOL due to increased intraoperative manipulation. Still, it was not statistically significant due to fewer patients with iris-claw. Further studies with more patients would be required to see any correlation between CCT and the IOL.

A vision-threatening complication after cataract extraction is DMD, which Scheie first described. In cases with clear cornea, DMD can be detected on slit-lamp examination, whereas, in cases with severe corneal edema, anterior segment optical coherence tomography (AS-OCT) remains the best imaging tool to diagnose its occurrence [2] conclusively. In the present study, all the patients who had corneal edema were examined with AS-OCT, and seven DMDs were noted with a mean corneal edema of 726.92 with an SD of 137.00 and a mean CCT of 497.55 with an SD of 49.70. All the DMD was found in trainee surgeons. This may be due to a lack of experience in identifying a detached DMD intraoperatively, which was effectively done by all the experienced surgeons, and reattaching the detached DM with the help of air injection.

The current study noted fifteen DMDs intraoperatively at the tunnel and the side port. As they were small DMDs, eight were found attached on the post-op day, one when seen on slit-lamp, and AS-OCT showed no evidence of DMD.

According to a study by Singhal D et al., AS-OCT has been used to confirm and classify DMD and helps decide the management plan [10]. Spontaneous reattachment of the DM occurs with conservative management in cases with small, peripheral, planar DMD with non-scrolled edges. Cases with nonplanar, central DMD, scrolled edges, and length > 2 mm should be managed surgically [11]. In the current study, five patients with a mean DMD < 100µm were successfully treated medically according to the HELP algorithm treatment protocol, and two patients with large DMD were treated with anterior chamber air injection. DM was attached successfully, and visual acuity improved with decreased stromal edema. In our study, all the patients with DMD were treated, medically and surgically, depending on size, with an anterior chamber air injection.

## Conclusion

Postoperative corneal edema following cataract surgery is a known complication that is usually resolved by medical management. More excellent surgical experience with a shorter duration of surgery and proper instrumentation was associated with reduced early postoperative corneal edema. Early detection and classification of DMD with the help of AS-OCT for those requiring medical and surgical management aid in recognizing the clinically relevant DMD, and thus, a timely switch to surgical management helps maintain vision in patients with DMD.
